# OLDER ADULT FALLS IN THE COMMUNITY: DOES UNSAFE HOME ENVIRONMENT HAVE A RISK ROLE?

**DOI:** 10.1093/geroni/igad104.1004

**Published:** 2023-12-21

**Authors:** Debasree Das Gupta, Uma Kelekar, Sidney Turner

**Affiliations:** Utah State University, Logan, Utah, United States; Marymount University, Arlington, Virginia, United States; Fors Marsh Group LLC, Falls Church, Virginia, United States

## Abstract

Hazardous environmental conditions are frequent in older adult homes and are linked to a majority of the older adult falls in the community. However it is unclear whether it is the presence/absence of home hazards per se, or the interaction between unsafe home conditions and physical functioning that is of salience to older adults’ fall risk. To address this gap, we conduct a mediation analysis using the Karlson–Holm–Breen methodology to estimate: 1) whether home disorder (peeling/flaking paint, pests: cockroaches/rodents, broken furniture/lamp, unsafe flooring/tripping hazards, room clutter) and unsafe bathroom features (absence of bathtub/shower stall, grab bar, seat in shower/tub, raised toilet) adversely associated with subsequent fall risk of older adults, and 2) what proportion of those influences were mediated through limitations in daily and instrumental activities of daily living (ADL/IADL). Using a nationally representative large sample of Medicare beneficiaries (≥65 years) living in the community who were respondents in the 2018-2019 National Health and Aging Trends Study (NHATS; n=2,599), we examined baseline home environment in 2018 and its relation with subsequent falls in 2019, after controlling for covariates. Results indicate while home disorder had both a direct (adjusted Odds Ratio [aOR]: 1.11, 95% Confidence Interval [CI]:1.01,1.22) and an indirect (aOR: 1.01; 95% CI: 1.00,1.02) effect on subsequent falls through limitations in ADL and IADL, unsafe bathroom features did not significantly predict subsequent fall risk of older adults. Given these findings, addressing home disorder through policy/housing assistance initiatives and educational programs highlighting home environmental safety would be essential.

